# A Case of Benign Gastrocolic Fistula With a Favorable Outcome Due to Preoperative Nutritional Therapy

**DOI:** 10.7759/cureus.80792

**Published:** 2025-03-18

**Authors:** En Amada, Tomohisa Kamo, Yoshihiro Watanabe

**Affiliations:** 1 Department of Surgery, Sonoda Daiichi Hospital, Tokyo, JPN

**Keywords:** benign gastric ulcer, endoscopic observation, gastrocolic fistula, nutritional treatment, preoperative diagnosis, surgical treatment

## Abstract

This case report details the management of a 70-year-old undernourished female patient presenting with a benign gastrocolonic fistula secondary to a massive gastric ulcer. Imaging confirmed the connection between the gastric ulcer and the transverse colon. The patient was placed on fasting and enteral nutritional management, resulting in a gradual improvement in her overall condition; however, the fistula persisted. Surgical intervention was performed on the 18th day post admission, comprising exploratory laparotomy, gastric resection, and partial colon resection. After surgery, the patient recovered without major complications.

## Introduction

Gastrocolic fistula is a direct communication between the stomach and the colon and is a relatively rare condition. In gastrocolic fistula, histopathological evidence shows communication between the stomach and the transverse colon, which can arise from both malignant and benign conditions [1]. The primary etiological factors for gastrocolic fistula are gastric or colonic adenocarcinoma, although lymphoma is a less prevalent cause. Clinically, gastrocolic fistula is often indicated by symptoms such as feculent vomiting, diarrhea, and significant weight loss [2]. Among the available diagnostic modalities, barium enterography is the preferred initial choice, while computed tomography (CT) scans and upper gastrointestinal radiography serve as alternative options. Laboratory findings may include anemia, leukocytosis, and electrolyte disturbances. Surgical intervention is usually the primary treatment, with timely surgery being crucial to prevent complications such as dehydration and malnutrition. The specific surgical approach is contingent on the patient's clinical situation and may necessitate resection of the affected areas.

In contemporary surgical practice, benign gastrocolic fistulas are rare due to enhanced medical management of gastric ulcer disease. Historically, these fistulas were more frequently associated with benign gastric ulcers; however, malignant causes have become increasingly prevalent [3]. Symptoms typically encompass non-specific complaints such as diarrhea, weight loss, and foul-smelling belching [4]. While barium enema remains a cornerstone for diagnosis, the effectiveness of CT in this specific context warrants further investigation [5].

Surgical management through en bloc resection, which is a surgical technique employed to excise a tumor along with its surrounding tissues in a single, contiguous piece, is regarded as the gold standard; nonetheless, recent guidelines advocate for prioritizing medical management when malignancy has been excluded. The underlying etiology of these fistulas may be intricate and could encompass conditions like microscopic lymphocytic colitis, which potentially elevates the risk of fistula formation [6]. Notably, despite advancements in medical therapies, instances of benign gastrocolic fistulas persist, even among patients receiving proton pump inhibitors.

 In this report, we present our experience with a benign gastrocolic fistula that proved resistant to conservative treatment.

## Case presentation

A female patient in her 70s, with a height of 149 cm and a weight of 39.0 kg, was admitted to our hospital following emergency transport due to black vomiting, fever, and melena, which had developed the day prior to her arrival. Her medical history included schizophrenia, for which she was currently receiving pharmacological treatment. Initial laboratory tests indicated increased inflammatory markers and hypoalbuminemia (Table 1).

**Table 1:** Results of blood sampling at initial examination

Parameter	Patient Value	Reference Range
Alkaline Phosphatase (ALP)	52 U/L	38 - 113 U/L
Lactate Dehydrogenase (LDH)	342 U/L	124 - 222 U/L
Total Protein (TP)	4.6 g/dL	6.7 - 8.3 g/dL
Albumin (Alb)	2 g/dL	3.8 - 5.2 g/dL
Aspartate Aminotransferase (AST)	27 U/L	10 - 40 U/L
Alanine Aminotransferase (ALT)	24 U/L	5 - 45 U/L
Gamma-Glutamyl Transferase (γ-GTP)	10 U/L	0.0 - 30 U/L
Cholinesterase (ChE)	71 U/L	200 - 452 U/L
Total Bilirubin	0.4 mg/dL	0.2 - 1.2 mg/dL
Creatine Kinase (CK)	88 U/L	40 - 150 U/L
Total Cholesterol	120 mg/dL	120 - 219 mg/dL
Triglycerides	161 mg/dL	30 - 149 mg/dL
Serum Amylase	68 U/L	40 - 122 U/L
Uric Acid	6.4 mg/dL	2.5 - 7.0 mg/dL
Sodium	134 mEq/L	137 - 147 mEq/L
Chloride	95 mEq/L	98 - 108 mEq/L
Potassium	4.3 mEq/L	3.5 - 5.0 mEq/L
Blood Urea Nitrogen (BUN)	33.5 mg/dL	8.0 - 20.0 mg/dL
Creatinine	0.93 mg/dL	0.47 - 0.79 mg/dL
C-reactive Protein (CRP)	4.35 mg/L	0.00 - 0.30 mg/L
Direct Bilirubin	0.1 mg/dL	0.0 - 0.2 mg/dL
Glucose	137 mg/dL	70 - 109 mg/dL
Hemoglobin A1c (HbA1c)	5.9%	4.6 - 5.2 %
White Blood Cell Count (WBC)	12200 /μL	3300 - 9000 /μL
Red Blood Cell Count (RBC)	226 10^4/μL	380 - 500 /μL
Hemoglobin	6.5 g/dL	11.5 - 15.0 /μL
Hematocrit	19.4%	34.8 - 45.0 %
Platelet Count	34.4 10^4/μL	14.0 - 34.0 10^4/μL
Mean Corpuscular Volume (MCV)	86 fL	85 - 102 fL
Mean Corpuscular Hemoglobin (MCH)	28.8 pg	28.0 - 34.0 pg
Mean Corpuscular Hemoglobin Concentration (MCHC)	33.5 g/dL	30.2 - 35.1 g/dL
Carcinoembryonic Antigen (CEA)	5 ng/mL	0.0 - 5.0 ng/mL
Cancer Antigen 19-9 (CA 19-9)	37 U/mL	0.0 - 37.0 U/mL

**Figure 1:**
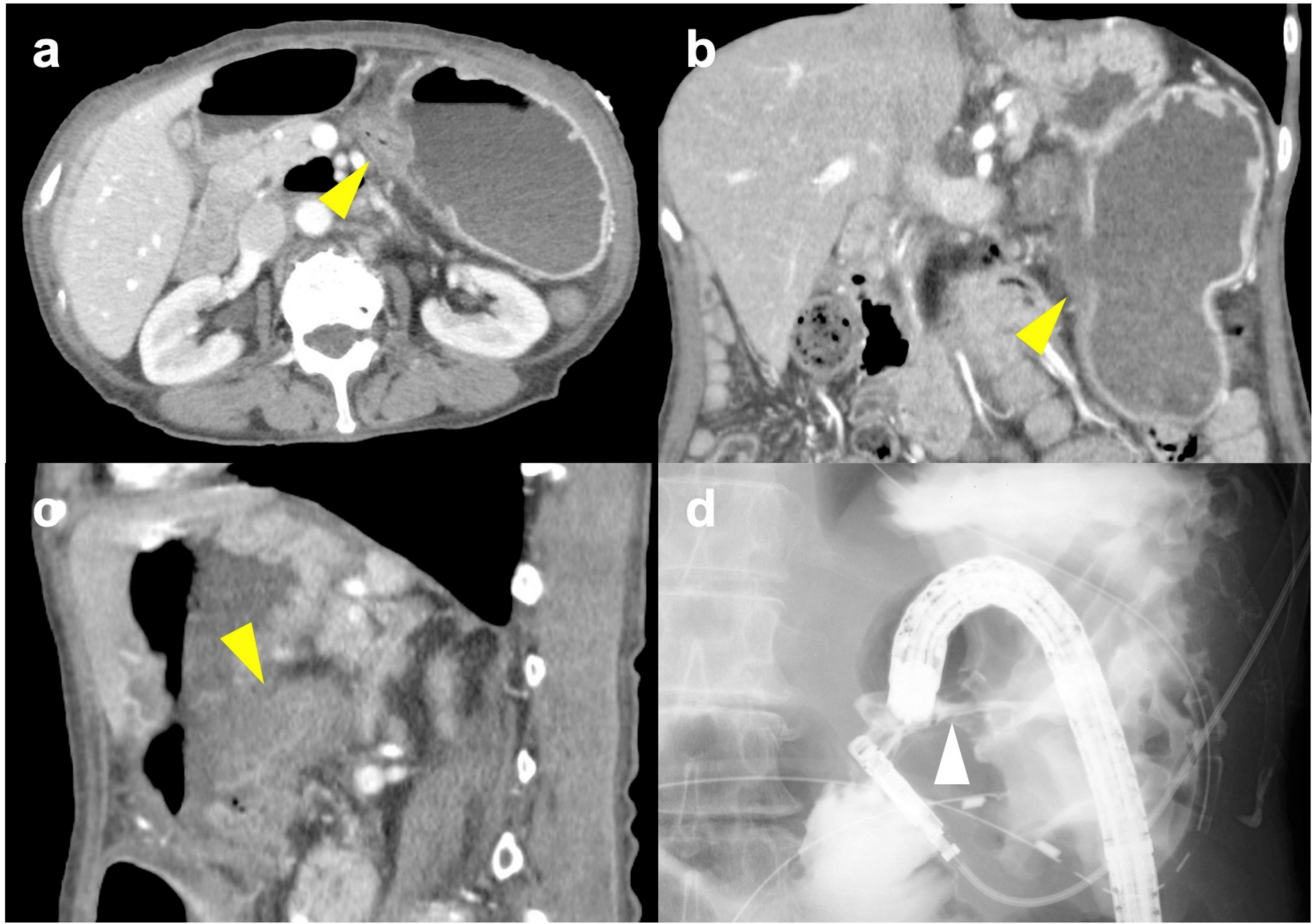
CT scan images and a fluoroscopic image of gastro-colic fistula. (a) Axial, (b) coronal, (c) sagital images from contrast-enhanced abdominal CT scan conducted at the time of admission, showing continuity between the mucosa of the transverse colon and the posterior wall of the mid-body of the stomach was demonstrated (yellow arrowhead). No clear signs of abscess formation were recognized. (d) Fluoroscopic image performed concurrently to lower gastrointestinal endoscopy, utilizing Gastrografin as the contrast agent from the colon, successfully visualizing the stomach through a fistula.

**Figure 2:**
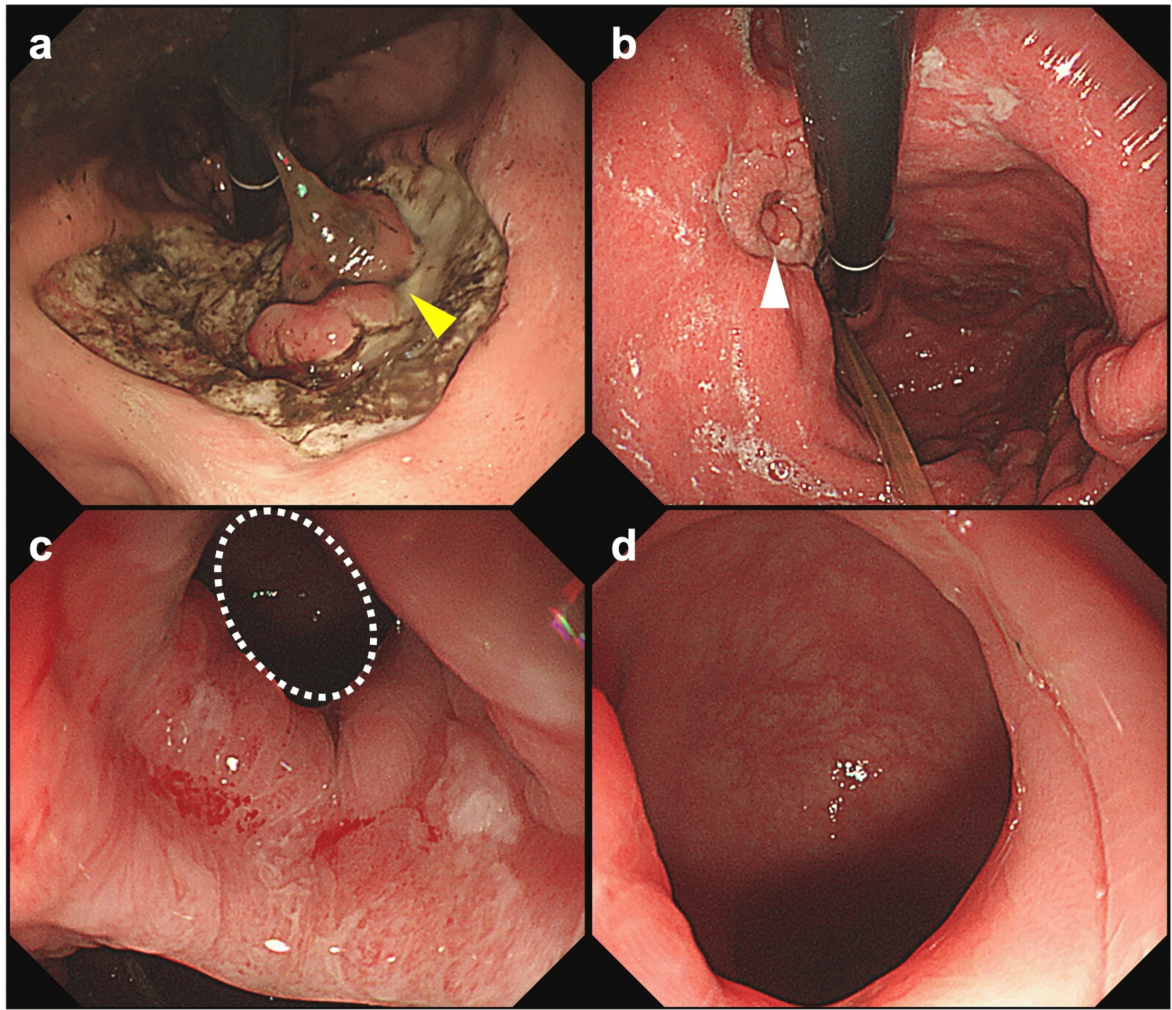
Upper gastrointestinal endoscopy images. (a) Image on the day of admission showing a massive ulcer extending from the upper to the mid-body of the stomach. A mucosal-like prominence is observed at the center of the ulcer base; (b) Image on 17th day of hospitalization showing the size of the ulcer base has reduced; however, the mucosal prominence at its center remains unchanged; (c) and (d): Image on 17th day of hospitalization. Upon closer examination, the lumen of the colon is visible through the center of this prominence.

A contrast-enhanced abdominal CT scan conducted at the time of admission demonstrated continuity between the mucosa of the transverse colon and the posterior wall of the mid-body of the stomach, with no clear signs of abscess formation (Figure 1). An upper gastrointestinal endoscopy performed on the day of admission revealed a massive ulcer extending from the upper to the mid-body of the stomach (Figure 2). Although there was no evidence of active bleeding, a mucosal-like prominence was observed at the center of the ulcer base.

**Figure 3:**
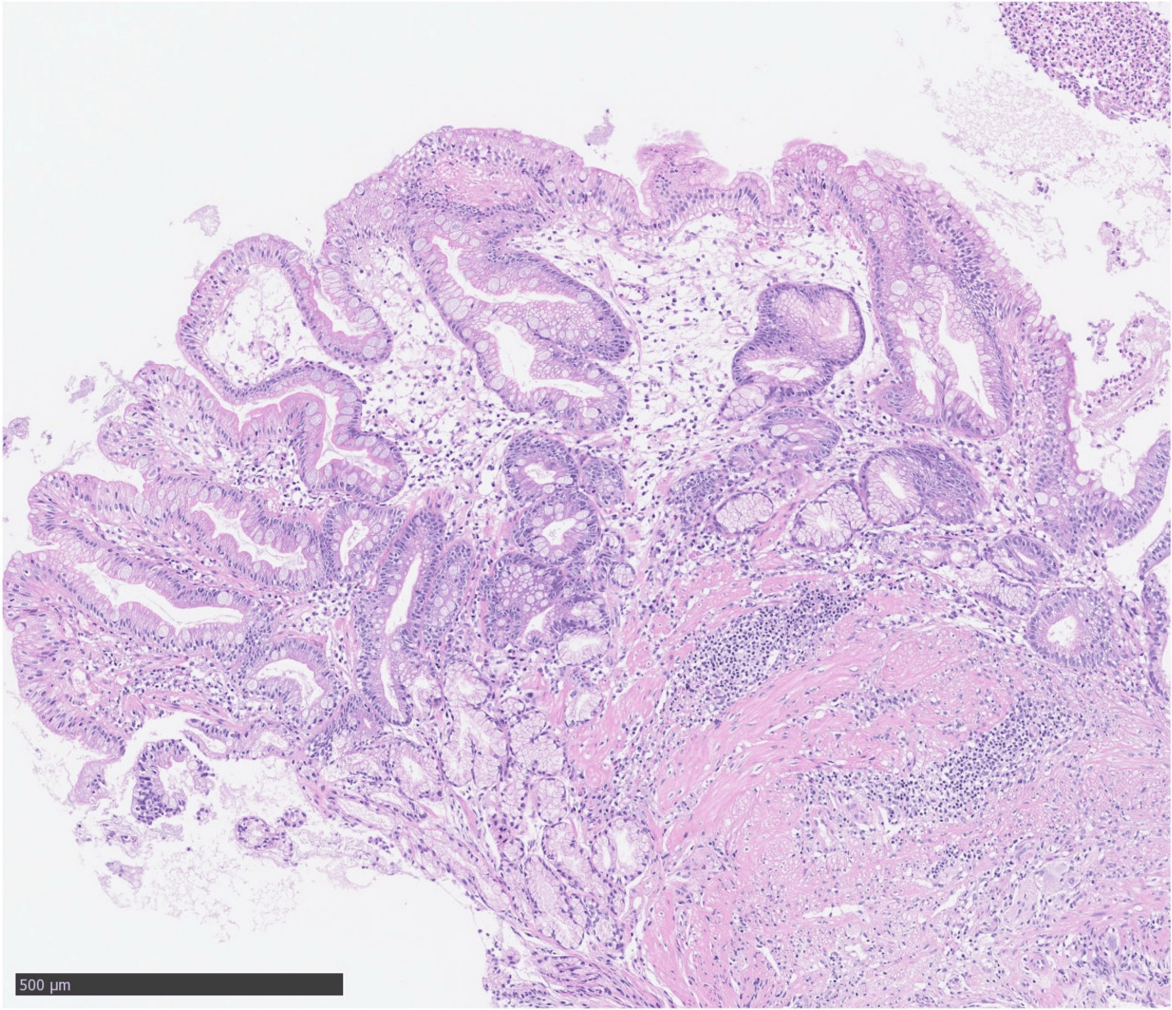
Histopathological examination of biopsy specimen taken from the ulcer base revealed prominent inflammatory granulation tissue with neutrophils and, in some fields, pseudopyloric glandular mucosa with intestinal epithelialization.

Following her admission, the patient was placed on prohibition of oral intake and enteral nutritional management using a double-lumen nasojejunal feeding and gastric aspiration tube, resulting in a gradual improvement in her overall condition. On the 17th day of hospitalization, a follow-up upper gastrointestinal endoscopy indicated a reduction in the size of the ulcer base; however, the mucosal prominence at its center remained unchanged (Figure 2). Upon closer examination, the lumen of the colon was visible through the center of this prominence. Simultaneously, a lower gastrointestinal endoscopy revealed significant angulation in the mid-transverse colon, which complicated scope passage. A fluoroscopic evaluation performed concurrently, utilizing Gastrografin as the contrast agent from the colon, successfully visualized the stomach through a fistula (Figure 1). The histopathological diagnosis based on a biopsy taken from the ulcer base was determined to be benign (Figure 3).

**Figure 4:**
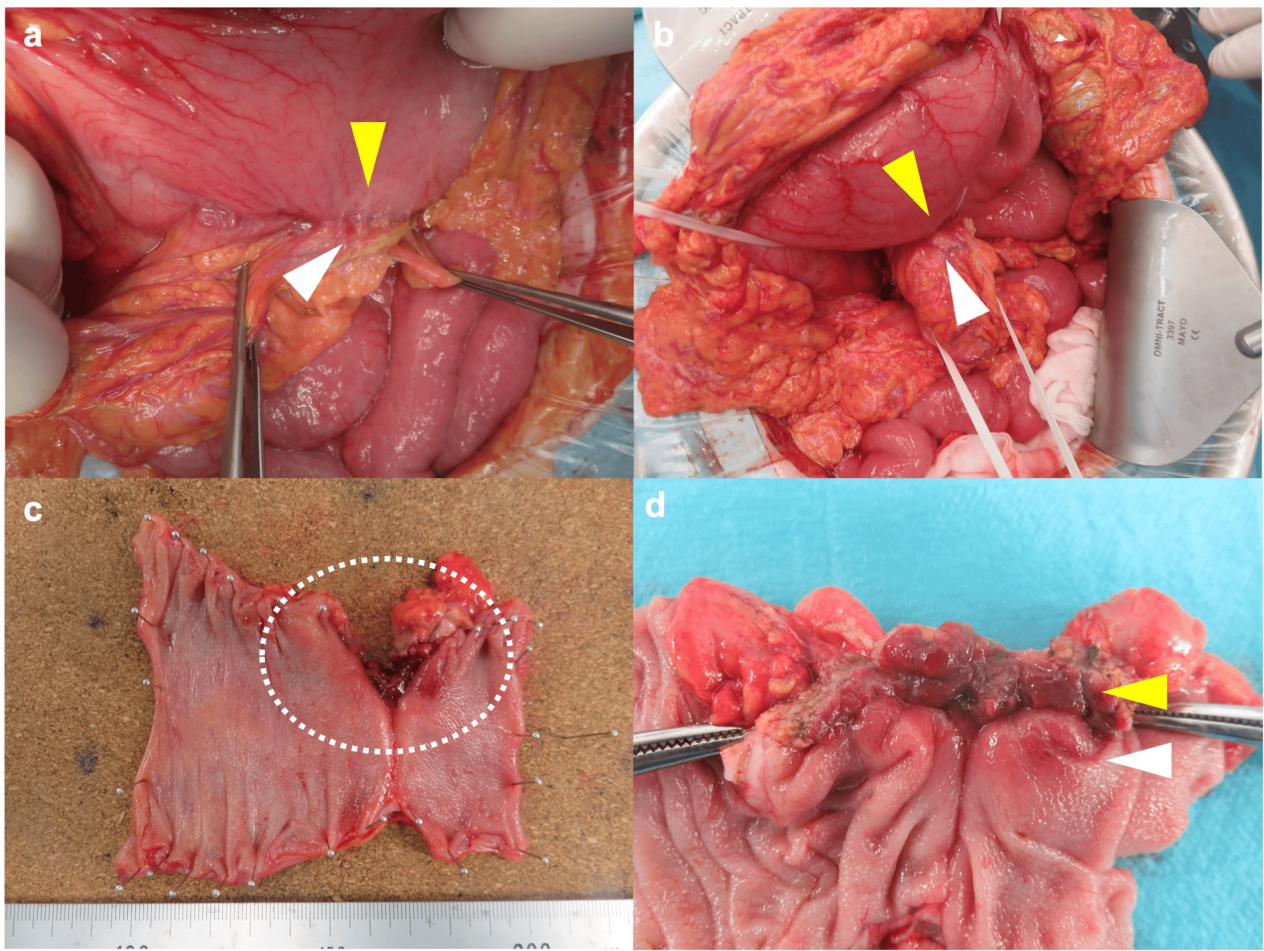
Intraoperative findings and images of the resected specimen. (a) and (b): On opening the omentum, a tightly adherent transverse colon (white arrowhead) was found on the posterior wall of the mid gastric lesser curvature (yellow arrowhead), which was considered to be a gastrocolic fistula. The left wall of the transverse colon was adherent to the stomach. (c) and (d): The resected specimen showed a fistula in which the gastric and colonic mucosa covered the lumen with continuity.

Given the diagnosis of a refractory benign gastrocolic fistula, a surgical approach was deemed necessary. On the 18th day of hospitalization, the patient underwent exploratory laparotomy, local gastric resection, partial resection of the transverse colon, and enterostomy creation (Figure 4).

On the eighth postoperative day, the patient exhibited delayed gastric emptying, classified as Clavien-Dindo Grade 2; however, her overall condition showed a progressive improvement, allowing for her transfer to a rehabilitation hospital on the 37th postoperative day. Subsequently, her oral intake stabilized, and the enterostomy was successfully removed three months following the surgery.

## Discussion

Benign gastrocolic fistula is an uncommon complication associated with gastric ulcer disease, with 108 documented cases reported by 1989 [7]. The frequency of case reports of this disease has decreased in recent years; a literature search revealed only a few case reports in the last decade [6,8-10]. Japan has seen a similar trend, with 16 case reports recognized before 2000 [11-25], compared to only five since then [26-30]. The rarity of benign gastrocolic fistulas is largely attributed to advancements in the medical management of gastric ulcers, particularly the widespread use of proton pump inhibitors.

Management strategies have evolved, with surgical intervention as the primary treatment approach [31]. Preoperative management has increasingly incorporated total parenteral nutrition and antibiotic bowel preparation to enhance patient outcomes. Mortality rates associated with surgical treatment have seen significant improvement, declining from 16% to 5.5% over the decades, due to advancements in nutritional support and sepsis management. Recent studies suggest that spontaneous closure rates of gastrocolic fistulas can reach up to 37% with appropriate nonsurgical interventions, and overall recovery rates may approach 93% under suitable management protocols [32].

A significant finding in this context is the favorable response to enteral nutritional management prior to surgical intervention. Nutritional therapy plays a crucial role in the management of patients with benign gastro-colic fistulas. Total parenteral nutrition has been used historically to prepare patients for surgery and promote spontaneous closure. However, recent evidence suggests that enteral nutrition should be prioritized when possible, with parenteral nutrition used as a supplement if necessary [33]. Nutritional requirements are known to vary based on fistula type and output, with high-output fistulas requiring increased energy and protein intake [34]. The lack of a standardized protocol necessitates individualized nutritional management.

While earlier studies have highlighted the importance of surgical procedures for the management of symptomatic cases, the initial conservative management strategy exhibited substantial improvement in the current patient. The surgical approach adopted, which included exploratory laparotomy and resection, is consistent with established treatment modalities. However, the expedited recovery observed in this case, when compared to historical data, may be indicative of advancements in perioperative care and rehabilitation practices. This case contributes to the understanding of benign gastrocolic fistulas and underscores the importance of integrated management strategies combining conservative and surgical approaches, which may lead to improved patient outcomes in future cases.

Previous reports have failed to identify cases of successful nutritional management with preoperative enteral nutrition. This may be due to the fact that the main symptom of the disease is attributed to poor nutritional status associated with diarrhea. The pathogenesis of this diarrhea is thought to be due to contamination of the upper gastrointestinal tract by fecal reflux into the stomach through the gastrocolic fistula, leading to fat indigestion due to the breakdown of bile salts, which eventually re-enters the colon, causing a catarrhal effect [35]. In this case, nutritional management was performed using a double-lumen nasojejunal feeding and gastric aspiration tube. This tube, which allows continuous aspiration of gastric contents, prevents the preoperative mixing of feces and nutritional products. This is thought to have led to more efficient nutritional management.

Surgery remains the first-line treatment for gastrocolic fistula. In previous reports, conservative treatment was successful in only five cases [30,36-39]. Despite recent advances in anti-ulcer drugs, the reason for the difficulty in healing with conservative treatment alone is thought to be that the granulation tissue forming the fistula is covered by the gastrointestinal mucosa during the healing process, including the surrounding inflammation caused by treatment.

## Conclusions

We experienced a case of benign gastrocolic fistula with poor nutritional status. Preoperative nutritional therapy was considered to have led to a positive outcome. The primary treatment for benign gastrocolic fistula is surgery, with preoperative parenteral nutrition contributing to a reduction in operative mortality. While total parenteral nutrition has traditionally been used, prioritizing enteral nutrition when feasible and supplementing with parenteral nutrition as required is emphasized. Nutrition plans should be tailored to individual patient needs, particularly with regard to fistula output, which affects energy and protein requirements. This case highlights the importance of comprehensive management strategies that integrate both conservative and surgical approaches, in line with advances in perioperative care, to improve patient outcomes.
